# A 3D cell culture system for bioengineering human neuromuscular junctions to model ALS

**DOI:** 10.3389/fcell.2023.996952

**Published:** 2023-02-14

**Authors:** Bita Massih, Alexander Veh, Maren Schenke, Simon Mungwa, Bettina Seeger, Bhuvaneish T. Selvaraj, Siddharthan Chandran, Peter Reinhardt, Jared Sterneckert, Andreas Hermann, Michael Sendtner, Patrick Lüningschrör

**Affiliations:** ^1^ Institute of Clinical Neurobiology, University Hospital Würzburg, Würzburg, Germany; ^2^ Institute for Food Quality and Safety, Research Group Food Toxicology and Alternative/Complementary Methods to Animal Experiments, University of Veterinary Medicine Hannover, Hannover, Germany; ^3^ Centre for Clinical Brain Sciences, University of Edinburgh, Edinburgh, United Kingdom; ^4^ UK Dementia Research Institute at University of Edinburgh, University of Edinburgh, Edinburgh, United Kingdom; ^5^ Anne Rowling Regenerative Neurology Clinic, University of Edinburgh, Edinburgh, United Kingdom; ^6^ Center for Regenerative Therapies Dresden, Technische Universität Dresden, Dresden, Germany; ^7^ Medical Faculty Carl Gustav Carus of TU Dresden, Dresden, Germany; ^8^ Translational Neurodegeneration Section “Albrecht-Kossel”, Department of Neurology, University Medical Center Rostock, Rostock, Germany; ^9^ Center for Transdisciplinary Neurosciences Rostock, University Medical Center Rostock, Rostock, Germany; ^10^ Deutsches Zentrum für Neurodegenerative Erkrankungen (DZNE) Rostock/Greifswald, Rostock, Germany

**Keywords:** NMJ–neuromuscular junction, motoneuron (MN), skeletal muscle, iPSC (induced pluripotent stem cells), 3D cell culture

## Abstract

The signals that coordinate and control movement in vertebrates are transmitted from motoneurons (MNs) to their target muscle cells at neuromuscular junctions (NMJs). Human NMJs display unique structural and physiological features, which make them vulnerable to pathological processes. NMJs are an early target in the pathology of motoneuron diseases (MND). Synaptic dysfunction and synapse elimination precede MN loss suggesting that the NMJ is the starting point of the pathophysiological cascade leading to MN death. Therefore, the study of human MNs in health and disease requires cell culture systems that enable the connection to their target muscle cells for NMJ formation. Here, we present a human neuromuscular co-culture system consisting of induced pluripotent stem cell (iPSC)-derived MNs and 3D skeletal muscle tissue derived from myoblasts. We used self-microfabricated silicone dishes combined with Velcro hooks to support the formation of 3D muscle tissue in a defined extracellular matrix, which enhances NMJ function and maturity. Using a combination of immunohistochemistry, calcium imaging, and pharmacological stimulations, we characterized and confirmed the function of the 3D muscle tissue and the 3D neuromuscular co-cultures. Finally, we applied this system as an *in vitro* model to study the pathophysiology of Amyotrophic Lateral Sclerosis (ALS) and found a decrease in neuromuscular coupling and muscle contraction in co-cultures with MNs harboring ALS-linked *SOD1* mutation. In summary, the human 3D neuromuscular cell culture system presented here recapitulates aspects of human physiology in a controlled *in vitro* setting and is suitable for modeling of MND.

## Introduction

Motoneurons (MNs) coordinate and control vertebrate movement by patterned electrical activity transmitted to skeletal muscle fibers to trigger their contraction. At neuromuscular junctions (NMJs), the MN axon terminates and interacts *via* chemical neurotransmitters with their target muscle cell. During neuromuscular transmission, MN activity causes the release of neurotransmitters from its terminals, which bind to postsynaptic receptors on the surface of their target cells to trigger a localized depolarization leading to muscle contraction (Li et al., 2018). NMJs have a unique morphology with a high degree of complexity on their presynaptic site. MN axons profoundly branch at their target site to contact many muscle fibers. These contact sites are highly specialized structures. The thin ending of the MN axon terminates as boutons from where the neurotransmitter is released (Li et al., 2018).

Although the principal organization of NMJs follows a similar blueprint, there are considerable inter-species differences (Jones et al., 2017; Boehm et al., 2020). A systematic comparative analysis between mouse and human NMJs revealed morphological differences, distinct synaptic proteomes, and distinct localization of the active zone proteins such as SNAP-25 (Jones et al., 2017). Compared to other vertebrate species, human NMJs are among the smallest known but with the most deeply infolded postsynaptic membrane (Jones et al., 2017; Slater, 2017; Boehm et al., 2020). From these morphological differences unique physiological properties arise, which need to be considered when studying pathological processes affecting the NMJ (Slater, 2017). In motoneuron diseases (MNDs) such as amyotrophic lateral sclerosis (ALS), NMJs become an early and vulnerable target, which represents the starting point of the pathological cascade leading to muscle atrophy and MN loss (Murray et al., 2010). This may explain the difficulty in translating studies based on ALS mouse models to clinical progress (Perrin, 2014; Clerc et al., 2016).

Thus, there is an urgent need to create cell culture models that reflect the human *in vivo* physiology as close as possible. So far, several approaches in generating models of neuromuscular circuits have been utilized (de Jongh et al., 2021). Initially, myofibers and induced pluripotent stem cell (iPSC)-derived MNs were co-cultured under 2D conditions to form NMJs (Demestre et al., 2015; Steinbeck et al., 2016). This was followed by work using compartmentalized 2D culture systems to ensure the maintenance of cell-type-specific microenvironments (Bellmann et al., 2019; Stoklund Dittlau et al., 2021). Such cell culture systems reflect the spatial separation of MN soma and axon terminal, which occurs *in vivo*. A more sophisticated 3D co-culture system was used to drive the maturation of the contractile apparatus reflected in the upregulation of the epsilon acetylcholine receptor (AChR) subunit (Afshar Bakooshli et al., 2019). The use of 3D muscle tissue in a compartmentalized system has also been described, but with a simplified protocol for creating muscle fibers (Osaki et al., 2018). Taken together, these studies show significant progress in the generation of 2D and 3D co-culture systems. However, the reproducibility of these methods and adaptation for MNs derived from different iPSC lines has not been shown.

The presented study aimed at establishing a 3D neuromuscular system with iPSC-derived MNs and myoblast-derived human skeletal muscle tissue. We decided to adopt the method described by Bakooshli et al. since it was the only study so far that provided evidence for adult NMJs *in vitro* (Afshar Bakooshli et al., 2019). We show an *in vitro* model that reproducibly generates the growth of MN axons within a 3D hydrogel resulting in the connection with myotubes and the formation of NMJs. Human skeletal myoblasts differentiate after 14 days to mature 3D muscle tissue. We confirmed muscle function by acetylcholine (ACh)-induced contraction and calcium imaging. Furthermore, we confirmed NMJ function by MN-induced muscle contraction with four different iPSC-lines. Using MNs derived from ALS patient iPSCs we generated co-cultures that showed a decrease in muscle contraction over time. These data demonstrated the applicability of this system to study human diseases that affect the NMJ. In summary, we present a human *in vitro* model to study NMJ physiology and dysfunction in the context of NMJ-affecting diseases like ALS.

## Materials and methods

### Human myoblast and human fibroblast culture

Primary human skeletal muscle myoblasts were purchased from Lonza (#CC-2580) and cultured according to Afshar Bakooshli et al. (2019) with minor modifications. Briefly, myoblasts were cultured in growth medium containing F-10 media (Gibco, #11550043), 20% fetal bovine serum (Gibco), 5 ng/mL basic fibroblast growth factor (bFGF) (PeproTech, #100-18B) and 100 μg/mL penicillin-streptomycin (Gibco, #15-140-122). Cells were cultured until 80%–90% confluency and used between passages 4–10 for the experiments. Primary human fibroblasts (Thermo Fisher, #C0045C) were cultured in growth medium until confluency. Myoblast and fibroblast medium was changed every other day.

### iPSC differentiation into neuronal progenitor cells (NPCs)

For the generation of 3D neuromuscular co-cultures, we used a previously reported human iPSC-line 34D6 (control iPSC-line #1) (Selvaraj et al., 2018), which was kindly provided by Prof. Chandran (University of Edinburgh). MNs were differentiated according to Reinhardt et al. (2013) or Kroehne et al. (2017) with some modifications. Briefly, iPSCs were expanded in mTeSR Plus (Stemcell Technologies, #05825) on Matrigel-coated (1:100) (Corning, 356234) dishes. 80%–90% confluent cells were split 1:5 using ReLeSR reagent (Stemcell Technologies, #05872). ROCK Inhibitor (10 µM) (StemMACS™ Y27632, Miltenyi Biotec, #130-106-538) was added for 24 h after splitting. For neuronal induction, mTeSR Plus medium was supplemented with 10 µM SB431542 (AdooQ BioScience, #A10826-50), 1 µM dorsomorphin homolog 1 (DMH1) (R&D Systems, #4126), 3 µM CHIR99021 (Cayman Chemical Company, #13122), and 0.5 µM Purmorphamine (PMA) (Cayman Chemical Company, #10009634). On day 2, the medium was changed to neuronal medium supplemented with the same small molecule supplements. Neuronal medium consisted of Neurobasal medium (Gibco, #21103049), Dulbecco’s modified Eagle’s medium F-12 (DMEM/F-12) (Gibco, #21331046), MACS NeuroBrew-21 (Miltenyi Biotech, #130-097-263), N-2 Supplement (Gibco, #17502048), 100 μg/mL Penicillin/Streptomycin/Glutamax (Gibco, #10378016). On day 4, the medium was changed to expansion medium consisting of neuronal medium supplemented with 3 µM CHIR99021, 0.5 µM PMA, and 150 µM Ascorbic acid (AA) (Sigma, #A92902). 80%–90% confluent cells were split and kept in suspension on uncoated dishes. Embryoid Bodies (EBs) formed from day 6 on in suspension and were selected and dissociated with a 1 mL pipette. Subsequently, cells were seeded on Matrigel-coated dishes. The resulting NPCs were split about once every week with Accutase (Thermo Fisher, #07920) and expanded for at least 15 passages to achieve pure NPC cultures. The medium was changed every other day.

### MN differentiation according to Reinhardt et al.

For ALS disease modeling, neuromuscular co-cultures were generated with the two previously reported human ALS iPSC-lines with a heterozygous R115G mutation and with a homozygous D90A mutation, respectively (Naujock et al., 2016; Gunther et al., 2022). ALS lines were kindly provided at NPC stage by Prof. Hermann (University Medical Center Rostock).

NPCs were cultured in expansion medium on Matrigel-coated dishes. For NPC differentiation into MNs, cells were cultured for 9 days in neuronal medium supplemented with 1 µM PMA. On day 2, 1 µM Retinoic acid (Stemcell Technologies, #72264) was added to the medium. The medium was changed every other day. For MN differentiation, the medium was switched after 9 days to neuronal medium supplemented with 10 ng/mL glia-derived neurotrophic factor (GDNF) (Alomone Labs, #G-240), 10 ng/mL brain-derived neurotrophic factor (BDNF) (PeproTech, #450-02), and 500 µM dibutyryl-cAMP (dbcAMP) (Stemcell Technologies, #73886).

### MN differentiation according to Kroehne et al.

NPCs were cultured for 10 passages in expansion medium with 0.5 µM smoothened agonist (SAG) (EMD Millipore, #566660) instead of 0.5 µM PMA on Matrigel-coated dishes. For NPC differentiation, cells were cultured for 6 days in neuronal medium supplemented with 0.5 µM SAG, 1 μM RA, 1 ng/mL GDNF, and 2 ng/mL BDNF. For MN differentiation, the medium was switched after 6 days to neuronal medium supplemented with 200 μM AA, 2 ng/mL GDNF and BDNF, 1 ng/mL transforming growth factor beta (TGFß3) (Sigma, #SRP3171), 200 µM dbcAMP, and 10 µM tert-Butyl(2S)-2-[[(2S)-2-[[2-(3,5-difluorophenyl)acetyl]amino]propanoyl]-amino]-2 phenylacetate (DAPT) (Cayman Chemical Company, #13197) for at least 14 days.

For a subset of co-culture experiments, we used MNs derived from the iPSC-line IMR90-4 (control iPSC-line #2), originally purchased from WiCell (Yu et al., 2007). Differentiation was performed as previously described (Schenke et al., 2020), based on protocols established by Kroehne et al. (2017). MNs were differentiated until day 6, cyropreserved, thawed and cultured as described above (on Matrigel dishes in neuronal medium supplemented with 200 μM AA, 2 ng/mL GDNF and BDNF, 1 ng/mL TGFß3, 200 µM dbcAMP and 10 µM DAPT) for at least 14 days.

### Self-manufactured 3D co-culture dishes

**FIGURE 1:**
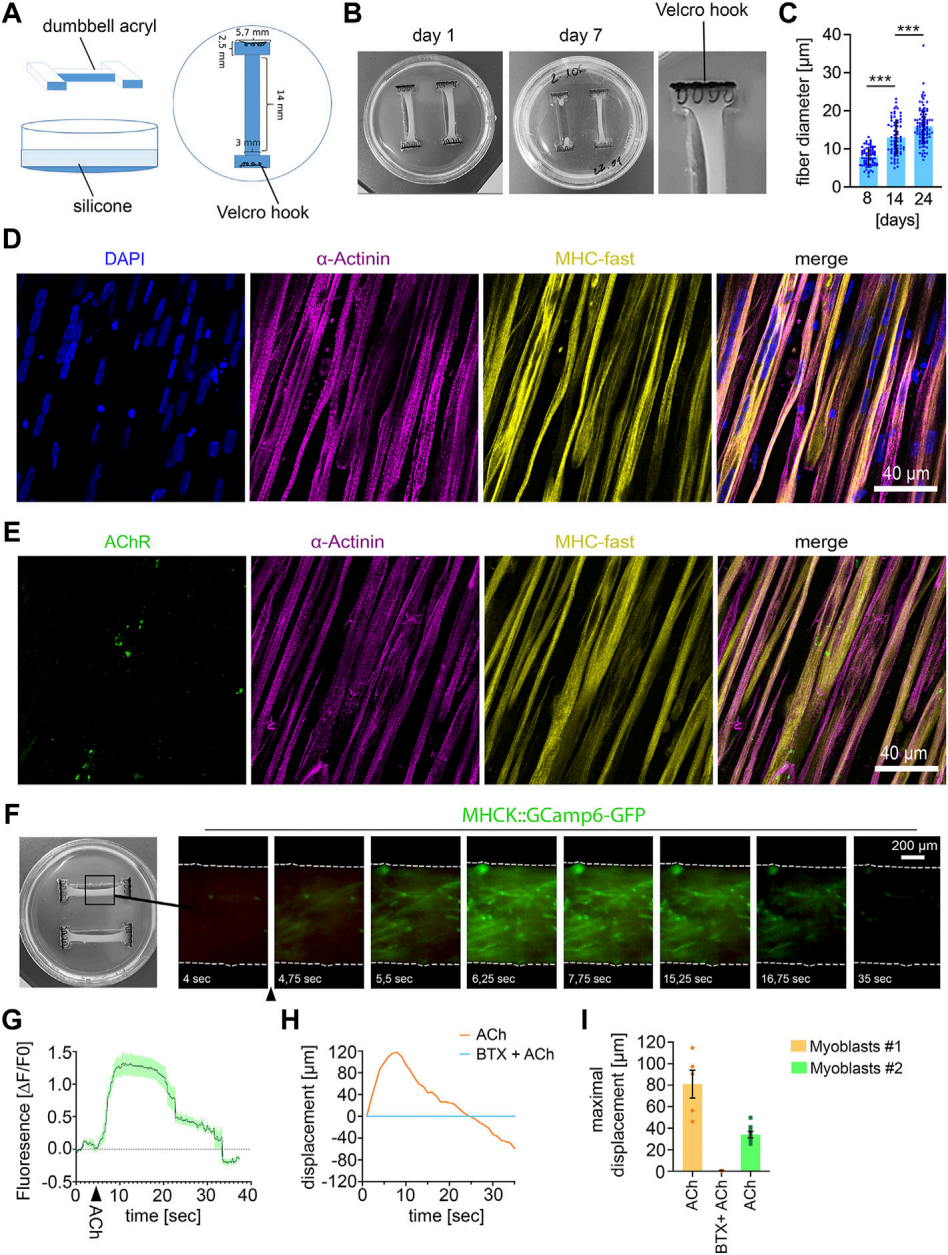
Establishing 3D human muscle tissue **(A)** Scheme of dumbbell-shaped template for microfabrication of the 3D culture dish. **(B)** Macroscopic images of the human muscle tissue with 1500000 myoblasts at the indicated culture time. The use of too high cell numbers (2000000 cells) lead to tearing of muscle fiber tissue (muscle tissue on the right side in the middle image). **(C)** Quantification of muscle fiber diameter. 8 days, *n* = 72; 14 days, *n* = 70; 24 days, *n* = 85; Mean ± SEM; each data point represents the diameter of an individual muscle fiber. One-way ANOVA, Tukey’s multiple comparisons test. **(D)** Immunocytochemical stainings of 21 days-old muscle fiber tissue against α-Actinin, Myosin Heavy Chain (MHC)- fast and DAPI. Scale bar: 40 μm. **(E)** Immunocytochemical stainings of 21 days-old muscle fiber tissue against Acetylcholine Receptors (AChR), α-Actinin and MHC fast. Scale bar: 40 μm. **(F)** Time lapse images of a representative calcium transient. Arrowhead indicates timepoint of ACh stimulation. **(G)** Representative calcium transient upon ACh treatment, each data point represents the mean of six ROIs. Mean ± SEM. **(H)** Quantification of the muscle fiber contraction. A representative curve for the muscle tissue displacement upon ACh treatment and upon pretreatment with BTX, followed by ACh stimulation is shown. **(I)** Quantification of the maximal muscle fiber displacement. Each data point represents the maximal displacement of an individual muscle fiber tissue. Myoblasts #1: ACh, *n* = 5; BTX + ACh, *n* = 2. Myoblasts #2: ACh, *n* = 8.

Polydimethylsiloxane (PDMS) (Sigma, #761036-5 EA) dishes were fabricated as described by Afshar Bakooshli et al. (2019) with some modifications. 3.5 cm Petri dishes (Sarstedt) were coated with 1.25 mL liquid PDMS and kept at 65°C for at least 3 h. Next, 1.75 mL PDMS were added, and two dumbbell shaped acrylic templates (middle channel dimensions = 14 mm by 2.75 mm; side chamber dimensions = 5.7 mm by 2.5 mm) (Figure 1A) were pressed into the PDMS next to each other. Air bubbles were removed within a vacuum chamber if necessary. After curing, acryl pieces were removed and 5.7 mm by 2.5 mm Velcro pieces were anchored on each channel side with liquid PDMS and cured again. Dishes were sterilized with 70% ethanol for 30 min and subsequently with 15 min UV light. Afterwards, the dishes were wrapped in parafilm and stored at room temperature (RT). PDMS dumbbell shape molds were incubated with 5% Pluronic acid (Thermo Fisher, #P6866) for at least 4 h at 4°C before applying the hydrogel.

### Generation of 3D skeletal muscle tissue

3D muscle tissues were generated as described by Afshar Bakooshli et al. (2019) with some modifications with two independent biopsies of skeletal myoblasts from Lonza (#CC-2580). For the hydrogel, 1500000 skeletal myoblasts, 75000 fibroblasts, 20% Geltrex (Gibco, #A1413302), 4 mg/mL Fibrinogen, 0.8 U Thrombin and DMEM/F-12 were mixed in a total volume of 200 μL, and filled into the PDMS channels completely covering the Velcro hooks. The hydrogel was incubated for 5 min at 37°C in the incubator prior to adding medium. 3D differentiation medium consisted of neuronal medium supplemented with 2% horse serum (Sigma, #H1138), 10 ng/mL Insulin (Merck, # I9278), 2 mg/mL 6-aminocaproic acid (ACA) (Sigma, #A2504), 2.5 μg/mL Amphotericin B (Sigma, #15290-026) and 50 ng/mL Agrin (R&D Systems, # 550-AG-100). The medium was changed every other day.

### 3D neuromuscular co-cultures

For the generation of 3D neuromuscular co-cultures, the hydrogel was prepared as described above and 500000 MNs were added to the hydrogel.

For neuromuscular co-cultures with MNs differentiated according to Reinhardt et al., MNs were used from day 9 of differentiation. 3D differentiation medium was supplemented with 10 ng/mL GDNF and BDNF and 500 µM dbcAMP. After 2 days, GDNF and BDNF concentrations were increased to 20 ng/mL. For neuromuscular co-cultures with MNs differentiated according to Kroehne et al. (control iPSC-line #1 derived MNs and control iPSC-line #2 derived MNs), MNs from differentiation day 6 were used and cultured in 3D differentiation medium supplemented with 200 μM AA, 2 ng/mL GDNF, 2 ng/mL CNTF (PeproTech, #450-13), and 2 ng/mL BDNF, 1 ng/mL TGFß3, 200 µM dbcAMP and 10 µM DAPT. After 2 days GDNF, CNTF, and BDNF concentrations were increased to 4 ng/mL.

The medium was changed every other day and co-cultures were cultured until the indicated time points for experiments.

### RNA isolation, cDNA synthesis, and quantitative real-time PCR (RT qPCR)

Total RNA was isolated with the NucleoSpin^®^ RNA kit (Macherey Nagel, #REF 740955.50). RNA was treated with DNase I (Thermo Fisher, #EN0521) to ensure the removal of genomic DNA. 100 ng total RNA was used for cDNA synthesis using the First Strand cDNA Synthesis Kit (Thermo Fisher, #K1612). For cDNA synthesis, random oligos were used. To ensure genomic DNA removal, for each sample set a control reaction was prepared without reverse transcriptase. cDNA was diluted 1:5 with water, and 1 µL was used for each qPCR reaction using the Thermo Scientific™ Luminaris HiGreen qPCR Master Mix (Thermo Fisher, # K0993) on a LightCycler 1.5 thermal cycler (Roche). The following primers were used: *HB9*: 5′-GCA​CCA​GTT​CAA​GCT​CAA​CA-3´ (Forward), 5′-TTC​TGT​TTC​TCC​GCT​TCC​TG-3´ (Reverse), *CHAT*: 5′-GAA​GAC​TGG​TGC​CCA​CCT​AC-3´ (Forward), 5′-GCA​TCC​TTC​AGG​AGC​AGA​A-3´ (Reverse), *SOX2* 5′-AAC​CCC​AAG​ATG​CAC​AAC​TC-3´ (Forward), 5′-CGG​GGC​CGG​TAT​TTA​TAA​TC-3´ (Reverse), *GAPDH* 5′-CTT​CGC​TCT​CTG​CTC​CTC​CTG​TTC​G-3´ (Forward), 5′-ACC​AGG​CGC​CCA​ATA​CGA​CCA​AAT-3´ (Reverse). For detection of *LHX3*, *HOXA2,* and *HOXA4* previously published primers were used (Reinhardt et al., 2013; Maury et al., 2015)*.*

### Immunocytochemical staining

Neuromuscular tissues and muscle tissues were transferred from 3D dishes to Eppendorf tubes, washed with phosphate-buffered saline (PBS), and fixed in 4% paraformaldehyde for 15 min at RT. Tissues were washed three times with PBS and incubated for 30 min in blocking solution containing 15% donkey serum and 0.3% Triton X-100 in TBS-Tween (TBST). After three washes with TBST, tissues were incubated with α -Bungarotoxin-Alexa-488 (α–BTX) (1:500) (Invitrogen, #B1601) for 30 min at RT and washed with TBST. Tissues were incubated overnight with primary antibodies diluted in 1:10 diluted blocking solution at 4°C. The next day, tissues were washed three times with TBST and incubated with appropriate fluorophore-conjugated secondary antibodies diluted 1:800 in 1:10 diluted blocking solution for 1 h at RT. Subsequently, tissues were washed three times with PBS and nuclei were counterstained with DAPI for 10 min and washed quickly with PBS. Squeeze preparations of the neuromuscular tissues and muscle tissue were mounted on glass slides with Aqua-Poly/Mount (Polysciences, #18606-20).

The same protocol was applied to stain iPSC-derived MNs differentiated according to Reinhardt et al. cultured for 14 days on Matrigel-coated coverslips.

16-bit images with 800 × 800–pixel or 1,024 × 1024–pixel resolution were acquired using an Olympus Fluoview 1,000 confocal microscope equipped with 20×, 40×, and 60× objectives. Images were processed with ImageJ.

The following primary antibodies were used: Anti- α- Actinin 2 (1:1,000, Sigma-Aldrich, ZRB1169, clone 1E11), anti- α- Actinin (1:1,000, Sigma-Aldrich, A7811, clone EA-53), anti-Myosin heavy chain human fast fibers (1:100, Developmental Studies Hybridoma Bank, A4.74, clone), anti-Tuj1 (1:1,000, Neuromics, MO15013), anti-Neurofilament heavy polypeptide antibody (1:1,000, Abcam, ab4680), anti-Synaptophysin-1 (1:1,000, Synaptic Systems, 101,004), anti-Islet-1 (1:500, Synaptic Systems, 406,003). Alexa-647-, Alexa-488- and Alexa-546-conjugated secondary antibodies were purchased from Jackson Immuno-Research Laboratories.

### Axon outgrowth measurements

To evaluate the axon stability in the neuromuscular co-cultures, the axon outgrowth in co-cultures was analyzed on day 9, day 21, and day 42. Co-cultures were fixed at the indicated time points as described above and stained with an anti-Neurofilament-H antibody (NF-H) (1:1,000, Abcam, ab4680). 16-bit images with 1,024 × 1024–pixel resolution were acquired using an Olympus Fluoview 1,000 confocal microscope with a 10× objective. At least four areas with an average size of 1.4 mm^2^ per sample were analyzed with ImageJ. To analyze the axon outgrowth, we determined the area covered with axons as visualized by the NF-H staining.

### Myofiber size analysis

For measuring the muscle fiber diameter, images of α-Actinin stained 3D muscle tissues were acquired with a 20x objective using an Olympus Fluoview 1,000 confocal microscope. The diameter of individual myofibers was manually measured using ImageJ.

### Muscle displacement measurements

Muscle contraction of 3D muscle tissue was imaged using an Olympus CKX41 microscope equipped with a LED illumination system (pE excitation system CoolLED) and an Olympus EP50 camera. 3 min videos were recorded with 10× magnification at 30 frames per second at 37°C. For stimulating muscle contraction, 100 µM ACh or 500 µM glutamate was directly applied to the culture dish as batch application. For control experiments, 10 min prior stimulation 1 μM BTX (Invitrogen, #B1601) or 60 nM tetrodotoxin (TTX) (Sigma, #T8024) were added, or 48 h prior stimulation 2 pM botulinum toxin (BoTN) (Miprolab, #3101-0010) was added. The muscle displacement was analyzed using ImageJ. At least two separate positions with highest contraction per muscle were individually measured. Fiber contraction at each position was traced per second and recorded as displacement per second. Longitudinal displacement was measured from the initial starting frame and displayed as movement in µm. Co-cultures derived from ALS iPSCs were evaluated in a blinded manner.

### Calcium imaging

For calcium imaging experiments a subset of myoblasts was transduced twice during expansion with an MHCK:GCamp6-GFP expressing lentivirus (pRRL-MHCK7-GCaMP6 was purchased from Addgene #65042) (Madden et al., 2015). 3 min videos were recorded using an Olympus CKX41 microscope equipped with a LED illumination system (pE excitation system CoolLED) and an Olympus EP50 camera with 10× magnification at eight frames per second at 37°C. Fluorescence changes over time were analyzed with ImageJ.

### Lentivirus production

Lentivirus was produced as previously described (Luningschror et al., 2017). Briefly, HEK 293T cells were transfected with the pRRL-MHCK7-GCaMP6 plasmid and the packaging plasmids using calcium phosphate. The medium was replaced 24 h after transfection and collected 24 h later. Subsequently, the virus was concentrated by ultracentrifugation.

### Statistics

Statistical analysis was performed in GraphPad Prism eight and statistical significance was considered at *p* < 0.05. Significance is designated as *, *p* < 0.05; **, *p* < 0.01, and ***, *p* < 0.001. The statistical test used to determine significant differences is described in the corresponding figure legend.

## Results

### Establishing 3D human muscle tissue

For microfabrication of the 3D muscle tissue culture dishes, we produced dumbbell-shaped acryl pieces as a first step (Figures 1A, B). Two of these acryl pieces were placed in a 3.5 cm cell culture dish and PDMS was poured into the dish to fabricate the dumbbell-shaped 3D muscle culture dishes. As anchoring points for the muscle tissue formation, Velcro pieces with nylon hooks were fixed on each end of the dumbbell-shaped channel with PMDS (Figure 1B).

For muscle tissue formation, myoblasts and fibroblasts were mixed with a fibrinogen/Geltrex hydrogel and seeded into the 3D dish. For optimization of muscle tissue formation, we tested different hydrogel to myoblast ratios (Supplementary Table S1). We observed that no muscle tissue was formed when cell numbers were too low in the hydrogel, whereas too many cells within the hydrogel lead to tearing of the muscle tissue (Figure 1B). 14 days after seeding the hydrogel-myoblast mixture, we observed the formation of 3D muscle tissue macroscopically. Next, we analyzed the fiber diameter and detected an increase over time suggesting the maturation of the muscle tissue (Figure 1C). Overall, myofibers aligned along the axes of the dish generating continuous muscle tissue. By immunostaining we could detect the muscle-specific markers sarcomeric α-Actinin, revealing the characteristic striated pattern, and expression of the fast myosin heavy chain (MHC). Furthermore, we detected the morphological formation of multinuclear myofibrils and AChRs clustering as visualized by α-Bungarotoxin (BTX) staining (Figures 1D, E).

To demonstrate the 3D muscle function, we analyzed the cytosolic calcium influx upon ACh stimulation (Figures 1F, G). Myoblasts were transduced with a lentivirus encoding the calcium sensor GCamp6-GFP under the muscle-specific MHCK7 promoter and subsequently used for muscle tissue formation. Upon stimulation with ACh, we detected a rapid fluorescence increase, which demonstrates a calcium influx into the cytosol (Figures 1F, G). Next, we analyzed muscle contraction by quantifying the tissue movement upon stimulation with ACh using live-cell imaging. ACh stimulation induced a fast contraction of the muscle tissue, which could be completely inhibited by blocking AChRs with BTX (Supplementary Video S1) (Figures 1H, I).

Given the limited passage number of primary myoblasts, we wondered if 3D muscle tissue can be reproducibly generated with myoblasts derived from a different muscle biopsy. Macroscopically, we did not observe any difference between both samples. Upon stimulation with ACh, we also observed a robust contraction of muscle tissue derived from the second batch of primary myoblasts (Figure 1I). However, quantification of the tissue movement revealed that the maximal muscle displacement caused by ACh was lower compared to the first batch of myoblasts.

### NMJ functionality in 3D neuromuscular co-cultures

**FIGURE 2:**
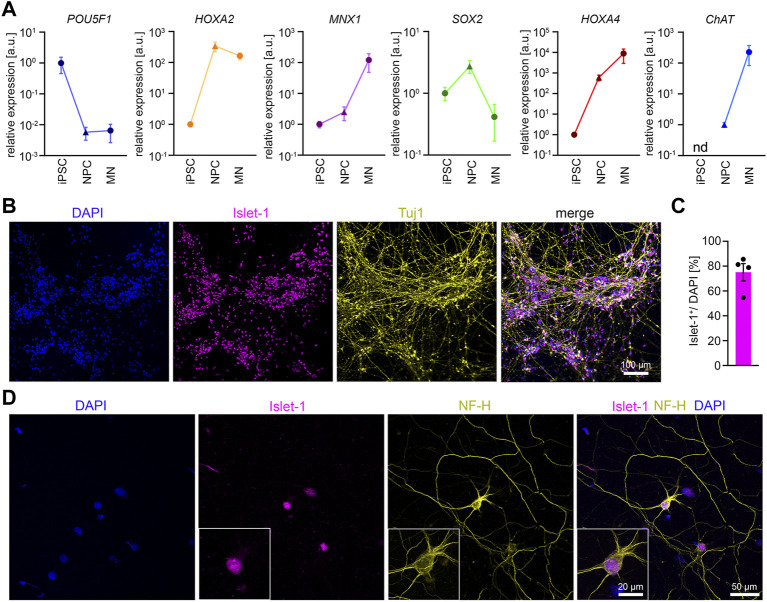
Differentiation of iPSCs into MNs **(A)** RT-qPCR analysis of the expression pattern of several marker genes during MN differentiation *n* = 5. Mean ± SEM. n.d., not detectable. **(B)** Representative low magnification image of iPSC-derived MNs stained for Islet-1 and Tuj1 after 14 days of maturation. **(C)** Quantification of Islet-1 positive cells. *n* = 5. Mean ± SEM. **(D)** Representative image of an individual iPSC-derived MN stained for Neurofilament Heavy Chain (NF-H) and Islet-1 after 14 days of maturation.

Next, we generated 3D neuromuscular co-cultures by adding iPSC-derived MNs to the hydrogel-myoblast mixture. For MN differentiation we utilized the previously established protocol by Reinhardt et al. (2013). To confirm a proper MN induction and differentiation, we performed RT-qPCR and immunocytochemical stainings (Figure 2). For RT-qPCR, samples were collected at different time points during differentiation. We analyzed the expression of *MNX1*, *ChAT*, *POU5F1*, *SOX2*, *HOXA2,* and *HOXA4* at the iPSC- and neural progenitor stage, and in MNs, 18 days after maturation (Figure 2A).

According to the ΔΔCT method we detected the following average fold changes in NPCs and MNs relative to the iPSC stage. The expression of the pluripotency marker *POU5F1* was reduced by 147 -fold in NPCs and 154-fold in MNs. The *SOX2* expression increased by 2.7-fold in NPCs but showed a downregulation by 10.7-fold from NPCs to MNs. The expression of the MN-specific transcription factor *MNX-1* was increased by 2.5-fold in NPCs, and by 120.7-fold in MNs. The expression of *CHAT* was not detectable in iPSCs. When normalized to NPCs, the *CHAT* expression increased by 226-fold in MNs. The expression of *HOX2A* increased by 339-fold from iPSCs to NPCs but deceased by 2.2-fold from NPCs to MNs. In contrast, during differentiation from iPSC to NPCs we detected a 604-fold increase in the expression of H*OX4A*, which further increased to 8800-fold from iPSCs to MNs. In summary, the data confirm neural induction and differentiation into spinal MNs. To examine the efficiency of MN differentiation, we stained MN cultures 14 days after maturation for Tuj1 and Islet-1. On average, roughly 75% of the cells stained positive for the MN marker Islet-1 (Figures 2B, C). MNs showed the expected polarity with one long axon and 3-4 dendrites as shown by Islet-1 and NF-H staining (Figure 2D).

**FIGURE 3:**
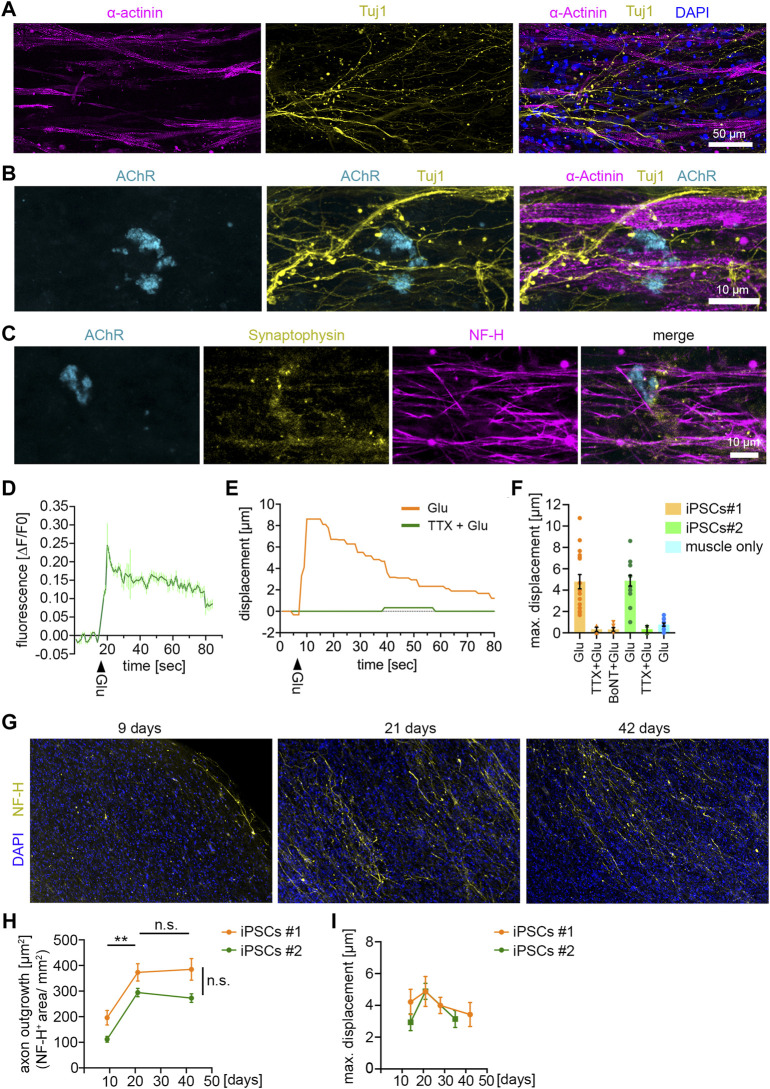
NMJ functionality in 3D neuromuscular co-cultures **(A)** Immunocytochemical stainings of a 21 days-old neuromuscular co-culture against Tuj1, α-Actinin and DAPI. Co-cultures were generated with MNs derived from control iPSC-line #1. MNs were differentiated according to Reinhardt et al. **(B)** Immunocytochemical stainings of a 21 days-old neuromuscular co-culture against AChR, Tuj1 and α-Actinin. **(C)** Immunocytochemical stainings of a 21 days-old neuromuscular co-culture against AChR, Synaptophysin-1 and NF-H. **(D)** Representative calcium transient upon glutamate treatment. Each data point represents the mean of seven ROIs. Mean ± SEM. Glu, glutamate. **(E)** Quantification of the muscle fiber contraction. A representative curve for the muscle tissue displacement upon glutamate treatment is shown. A second curve shows the muscle displacement after pretreatment with TTX, followed by glutamate stimulation. TTX, tetrodotoxin. Glu, glutamate. **(F)** Quantification of the maximal muscle fiber displacement after 21 days. Two independent iPSC lines were used to generate MNs, which were subsequently used for the co-cultures. Each data point represents the maximal displacement of an individual muscle fiber tissue. Mean ± SEM. iPSCs#1: Glu, *n* = 17; TTX + Glu, *n* = 4; BoNT + Glu, *n* = 6. iPSCs#2: Glu, *n* = 15; TTX + Glu, *n* = 2. Muscle only, *n* = 13. **(G)** Representative low magnification images of co-cultures stained for NF-H and DAPI at the time points indicated. **(H)** Quantification of the axon outgrowth. Two independent iPSC lines were used to generate MNs, which were subsequently used for the co-cultures. iPSCs#1: Day 9, *n* = 7; Day 21, *n* = 4; Day 42, *n* = 11. iPSCs#2: Day 9, *n* = 7; Day 21, *n* = 8; Day 42, *n* = 10. **(I)** Quantification of the maximal muscle fiber displacement. Co-cultures were stimulated with glutamate and the tissue movement was analyzed at the time points indicated. iPSCs#1: Day 14, *n* = 13; Day 21, *n* = 10; Day 28, *n* = 16; Day 42, *n* = 7. iPSCs#2: Day 14, *n* = 5; Day 21, *n* = 15; Day 35, *n* = 6.

After 14 days of co-culturing, we observed axons growing in between muscle fibers as shown by Tuj1 and α-Actinin staining (Figure 3A). Furthermore, we detected MN terminals close to AChR^+^ clusters, indicating the formation of NMJs (Figure 3B). NMJ formation was further confirmed by Synaptophysin-1 and AChR staining as pre- and post-synapse marker (Figure 3C). Pre-synaptic structures co-localized at MN axons (branches) shown by Synaptophysin-1 and NF-H staining (Figure 3C).

To test the functionality of the NMJs, we stimulated co-cultures with glutamate to induce MN activity. Upon firing ACh is released from the MN terminals which triggers depolarization on the postsynaptic side in the muscle cells. Using GCamp6-GFP-expressing muscle tissue we observed an increase of free calcium in the cytosol of muscle cells upon glutamate stimulation (Figure 3D), suggesting functional signal transduction from MN to muscle. Next, we analyzed the muscle contraction upon glutamate stimulation in co-cultures generated with MNs derived from two independent iPSC-lines (Figure 3F). Glutamate treatment triggered a muscle tissue contraction (Supplementary Video S2) (Figures 3E, F), that could be completely abolished by blocking the activity of voltage-gated sodium channels using TTX (Figures 3E, F). To confirm that the muscle contraction was induced by presynaptic MN activity, and not by activation of postsynaptic glutamate receptors (Mays et al., 2009), we specifically inhibited the presynaptic activity with BoTN. Indeed, pretreatment of co-cultures with BoTN blocked the glutamate-induced muscle contraction (Figure 3F). Moreover, we did not detect any significant glutamate-induced contraction in muscle tissues grown without MNs (Figure 3F). Taken together, these data show that functional NMJs are generated between iPSC-derived MNs and muscle tissue.

Next, we wondered about the stability of this system and maintained MNs and muscle tissue for up to 6 weeks in culture (Figures 3G–I). We analyzed the axon growth after 9, 21, and 42 days by NF-H staining (Figures 3G, H). We found that the axon growth peaked at day 21. From day 21–42 axon growth stagnated, but the axon integrity remained stable, suggesting that they reached their target during this period (Figures 3G, H). To analyze whether the functionality of connections between MNs and muscle tissue remained stable, we stimulated the co-cultures with glutamate at different time points for up to 6 weeks (Figure 3I). The glutamate-induced muscle contraction peaked at 21 days and started to decline up to 42 days. However, these differences were minor and statistically not significant, indicating that the connections between muscle and MNs remained stable during the investigated period.

### ALS disease modeling

**FIGURE 4:**
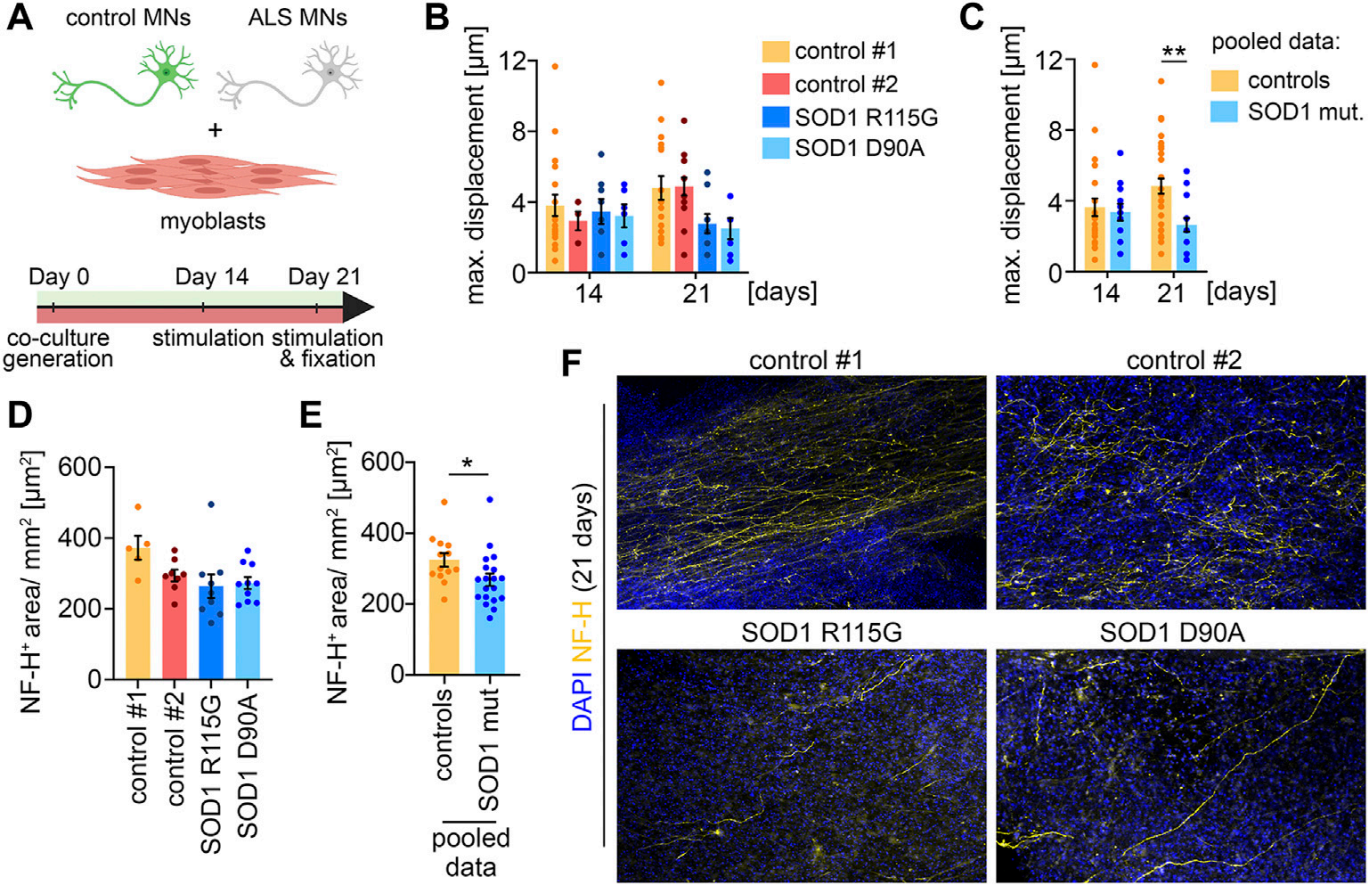
ALS disease modeling **(A)** Scheme of the experimental setup. **(B)** Quantification of the maximal muscle fiber displacement. Co-cultures were stimulated with glutamate after 14 and 21 days in culture and the tissue movement was analyzed. Control#1: Day 14, *n* = 20; Day 21, *n* = 17. Control#2: Day 14, *n* = 5; Day 21, *n* = 15. *SOD1* ^*+/R115G*^: Day 14, *n* = 8; Day 21, *n* = 9. *SOD1* ^*D90A*/*D90A*^: Day 14, *n* = 6; Day 21, *n* = 8. **(C)** Pooled data of the quantifications shown in **(B)**. Controls (control#1 & control#2): Day 14, *n* = 25; Day 21, *n* = 32. SOD1 mut. (*SOD1* ^*+/R115G*^ & *SOD1* ^*D90A*/*D90A*^): Day 14, *n* = 22; Day 21, *n* = 17. Two-tailed *t*-test. **(D)** Quantification of the axon outgrowth after 21 days. Control#1: *n* = 5. Control#2: *n* = 8. *SOD1* ^*+/R115G*^: *n* = 10. *SOD1* ^*D90A*/*D90A*^: *n* = 10. **(E)** Pooled data of the quantifications shown in **(D)**. Controls (control#1 & control#2): *n* = 13. SOD1 mut. (*SOD1* ^*+/R115G*^ & *SOD1* ^*D90A*/*D90A*^): *n* = 20. Two-tailed *t*-test. **(F)** Representative low magnification images of co-cultures stained for NF-H and DAPI after 21 days.

To demonstrate that the co-culture system is applicable as a disease model, we differentiated two iPSC lines derived from ALS patients with *SOD1* mutations to MNs, which were subsequently co-cultured with muscle tissue (Figure 4A). One of the patients carried a heterozygous R115G mutation. The other patient carried a homozygous D90A mutation. Both iPSC lines have previously been characterized (Naujock et al., 2016; Gunther et al., 2022). Macroscopically, co-cultures with ALS MNs were indistinguishable from healthy controls. After 14 days in culture, co-cultures with MNs derived from ALS iPSCs showed a similar glutamate-induced muscle contraction to control cultures, indicating a functional integration of the ALS MNs (Figures 4B, C). Interestingly, we observed a decrease in muscle contraction in co-cultures generated with ALS MNs after 21 days. This effect was observed with both *SOD1* mutations (Figure 4B) and reached statistical significance when the results from both controls and the ALS cultures were pooled (Figure 4C). After 21 days, the co-cultures were fixed and stained for NF-H to analyze the axon stability (Figures 4D–F). In correlation with the decrease in muscle contraction, we detected a reduced axonal coverage of the muscle tissues in ALS co-cultures (Figures 4E, F). Taken together, these data suggest that MNs carrying SOD1 mutation successfully integrate into the system, but start to display a pathological phenotype at a later time point.

## Discussion

Here we adapted, validated, and applied a previously described method to co-culture 3D human skeletal muscle tissues together with human iPSC-derived MNs. We used this system to generate co-cultures with MNs harboring ALS-linked *SOD1* mutations and found that these MNs were able to functionally integrate into the culture but started to show signs of a pathological phenotype at a later stage. Our results show the successful generation of functional human NMJs *in vitro,* which can be used as a disease model to study the pathophysiology of MNDs like ALS.

In this study, we generated functional muscle tissue with primary myoblasts derived from two different biopsies. Whereas both batches of primary myoblasts robustly formed well-structured contractile muscle tissue, we observed a batch-to-batch variability in the strength of muscle contraction. These data show that biopsy-derived myoblasts are a reliable source for 3D muscle tissue generation that is capable to form functional connections with MNs in neuromuscular co-cultures. However, the limited passage number and batch—to-batch variability represent disadvantages of primary myoblasts as the starting cell population. Nagashima et al. (2020) recently employed the human immortalized human myogenic cell line Hu5/KD3 to generate contractile muscle tissues. Testing Hu5/KD3 cells for their capability to form muscle tissue that functionally connects with MNs will be an interesting future direction to overcome the limitation of primary myoblasts.

When co-cultured with MNs, we observed a robust contraction of the muscle tissue upon glutamate stimulation. This contraction could be completely abolished by TTX and BoNT. Moreover, glutamate did not induce any contraction in muscle tissue cultured without MNs. Thus, glutamate induces muscle contraction by triggering MN activity, and not by activation of postsynaptic glutamate receptors on the surface of muscle cells (Mays et al., 2009). In summary, these data demonstrate the functional connection between MNs and muscle tissue.

However, on the morphological level, bioengineered human NMJs differ from their *in vivo* counterparts. Two recent comparative studies characterized human NMJs *in vivo* (Jones et al., 2017; Boehm et al., 2020). Compared to other vertebrate species human NMJs are small, with thinner pre-terminal axons, more rudimentary nerve terminals, and distinctive endplates formed like coin-shaped patches (referred to as “nummular”) (Jones et al., 2017; Boehm et al., 2020). However, they show the highest extent of postsynaptic folding among vertebrate species (Slater, 2008). In contrast, *in vitro* only contacts between MN axons and muscle fibers without any typical “NMJ-like” morphology have been described so far (Steinbeck et al., 2016; Osaki et al., 2018; Afshar Bakooshli et al., 2019; Bellmann et al., 2019; Stoklund Dittlau et al., 2021). We also observed this phenomenon in our study. To the best of our knowledge, NMJs with typical morphological features such as the postsynaptic folding have not been reported yet.

Several aspects could be considered to improve NMJ maturation. During embryonic development initial connections between MNs and myotubes are unspecialized without showing typical NMJ morphology, yet. However, these first contacts are capable of rudimentary synaptic transmission (Sanes and Lichtman, 1999). During maturation, the NMJ morphology undergoes dramatic changes. If *in vitro* generated NMJs represent an early developmental stage, a prolonged culture period could play a crucial role to ensure NMJ maturation. Our data indicate that neuromuscular co-cultures are viable and functional for at least up to 6 weeks. Especially when considering the different time periods for embryonic development of most model organisms compared to humans, it will be an exciting future direction to test whether longer culture periods contribute to NMJ maturation.

In addition to the culture period, repeated stimulation is most likely required to support NMJ maturation. This could be achieved by enriching the culture medium with e.g., NMDA and/or AMPA receptor agonists. A different strategy could be a repeated optogenetic or electrical stimulation of MNs and/or muscle tissue.

Another important aspect to improve NMJ maturation and maintenance are defined medium components to co-culture different cell types in 3D. Besides Agrin, several other molecules like fibroblast growth factor, HB-GAM/pleitrophin, laminin beta 2, and midkine have been reported to promote NMJ formation (Sanes and Lichtman, 1999).

During embryonic and postnatal NMJ maturation *in vivo*, terminal Schwann cells play a fundamental role in addition to MNs and muscle cells. In a recent publication with self-organizing human neuromuscular organoids (Faustino Martins et al., 2020) the differentiation of terminal Schwann cells at NMJs was reported, pointing to the importance of this cell type. Schwann cells cap the nerve terminal, supporting its morphology and maintenance (Alvarez-Suarez et al., 2020; Faustino Martins et al., 2020). On the muscle side, Schwann cells enhance myotube growth and size and number of AChRs (Horner et al., 2021). Therefore, the inclusion of Schwann cells in the co-culture would be an interesting approach for the improvement of this system.

To validate the developmental stage of bioengineered NMJs, electrophysiological measurements are needed to determine the physiological properties of the MNs. However, the size and 3D structure of the co-culture make it difficult to identify innervated muscle fibers and the corresponding MNs that make synaptic contact to them. MNs and muscle fibers are randomly mixed, and fluorescent-labeled MNs can only get precisely identified at the confocal microscope. Recently, an elegant Rabies virus-based retrograde tracing approach has been applied to a neuromuscular co-culture system (Bellmann et al., 2019). Utilizing such an approach could overcome the challenge of identifying innervated muscle fibers.

To test the applicability of this system, we generated and analyzed co-cultures with MNs derived from ALS patient iPSCs. We show that these MNs functionally integrate into the system but start to show signs of a pathological phenotype at 21 days of culture. These data demonstrate that the neuromuscular co-culture system presented here is suitable for human *in vitro* disease modeling of the NMJ. Our data are in agreement with a recent study with utilized sensorimotor organoids to model ALS *in vitro.* Among different ALS mutations, organoids carrying a *SOD1* ^*G85R*^ mutation were analyzed and an impairment at the NMJ level was identified, as shown by contraction and immunocytochemical measurements (Pereira et al., 2021). Future studies employing human *in vitro* models of NMJs may help to develop and validate new mechanistic hypotheses for early pathogenesis in neuromuscular diseases. For ALS in particular, NMJs appear as an early and vulnerable target and provide the most important pathological and functional disease readouts (Murray et al., 2010).

Taken together, we report the generation of a functional 3D neuromuscular co-culture system using myoblast-derived muscle tissue and iPSC-derived MNs. This co-culture represents a valuable tool for modeling NMJ-affecting human diseases, but also for studying human NMJ development.

## Data Availability

The raw data supporting the conclusion of this article will be made available by the authors, without undue reservation.

## Supplementary material

Supplementary Video S1 3D muscle conraction upon 100 μM ACh stimulation.

Supplementary Video S2 3D co-culture contraction upon 500 μM glutamate stimulation.
